# Protein carbamylation is associated with increased mortality and CKD progression in patients with CKD: results from the EQUAL study

**DOI:** 10.1093/ckj/sfaf302

**Published:** 2025-10-15

**Authors:** Tilla C Folttmann, Antje M Haas, Nicholas C Chesnaye, Kitty J Jager, Fergus J Caskey, Maria Pippias, Friedo W Dekker, Merel van Diepen, Marie Evans, Claudia Torino, Antonio Vilasi, Maciej Szymczak, Christoph Wanner, Anders H Berg, Christiane Drechsler

**Affiliations:** Department of Clinical Research and Epidemiology, Comprehensive Heart Failure Center, University Hospital of Würzburg, Würzburg, Germany; Medizinische Klinik I, Universitätsklinikum Schleswig-Holstein, Lübeck, Germany; Department of Clinical Research and Epidemiology, Comprehensive Heart Failure Center, University Hospital of Würzburg, Würzburg, Germany; ERA Registry, Department of Medical Informatics, Amsterdam UMC, University of Amsterdam, Amsterdam, the Netherlands; Amsterdam Public Health Research Institute, Quality of Care, Amsterdam, the Netherlands; ERA Registry, Department of Medical Informatics, Amsterdam UMC, University of Amsterdam, Amsterdam, the Netherlands; Amsterdam Public Health Research Institute, Quality of Care, Amsterdam, the Netherlands; Population Health Sciences, Bristol Medical School, University of Bristol, Bristol, UK; Population Health Sciences, Bristol Medical School, University of Bristol, Bristol, UK; Renal Unit, Bristol Trust, Bristol, UK; Department of Clinical Epidemiology, Leiden University Medical Center, Leiden, the Netherlands; Department of Clinical Epidemiology, Leiden University Medical Center, Leiden, the Netherlands; Renal unit, department of Clinical Intervention and technology (CLINTEC), Karolinska Institutet and Karolinska University hospital, Stockholm, Sweden; Department of Clinical Sciences Intervention and Technology, Karolinska University Hospital Huddinge, Stockholm, Sweden; IFC-CNR, Clinical Epidemiology and Pathophysiology of Renal Diseases and Hypertension, Reggio Calabria, Italy; IFC-CNR, Clinical Epidemiology and Pathophysiology of Renal Diseases and Hypertension, Reggio Calabria, Italy; Department of Nephrology and Transplantation Medicine, Wroclaw Medical University, Wroclaw, Poland; Department of Clinical Research and Epidemiology, Comprehensive Heart Failure Center, University Hospital of Würzburg, Würzburg, Germany; Department of Pathology and Laboratory Medicine, Cedars-Sinai Medical Center, Los Angeles, CA, USA; Department of Clinical Research and Epidemiology, Comprehensive Heart Failure Center, University Hospital of Würzburg, Würzburg, Germany; Interdisciplinary Center for Palliative Medicine, University Hospital Wuerzburg, Würzburg, Germany

**Keywords:** carbamylated albumin, CKD, EQUAL, hyperuraemia, protein carbamylation

## Abstract

**Background and hypothesis:**

Urea accumulated in CKD patients’ blood can spontaneously decompose into reactive isocyanate and bind to plasma proteins in a reaction called carbamylation. Recent studies suggest a direct link between protein carbamylation and the pathogenesis of cardiovascular events and mortality in dialysis patients. We investigated whether carbamylation of albumin (C-Alb) is associated with increased mortality, major adverse cardiovascular events, and need for dialysis in older patients with advanced CKD.

**Methods:**

The European Quality Study (EQUAL) is a multicentre prospective cohort study. CKD patients aged 65 or older with advanced CKD (eGFR ≤20 ml/min/1.73 m²) not on kidney replacement therapy were followed up for 5 years. In a subgroup of 1117 patients, C-Alb was measured at baseline using combined liquid chromatography and mass spectrometry. Multivariable analyses were adjusted for important confounders.

**Results:**

Mean C-Alb was 13.5 ± 6.5 mmol/mol. Men had higher C-Alb values than women, as well as patients with chronic heart failure compared to patients without. C-Alb correlated positively with age, creatinine, urea, and negatively with eGFR, but not with total albumin. Each unit increase of log-transformed C-Alb was associated with increased risk of overall mortality (adj. HR 1.92, 95%CI 1.40–2.64) and start of dialysis therapy (adj. HR 1.59, 95%CI 1.21–2.09).

**Conclusion:**

In older advanced CKD patients not on dialysis, increased levels of C-Alb were associated with higher mortality and need for dialysis.

## Graphical Abstract

**Graphical Abstract:**
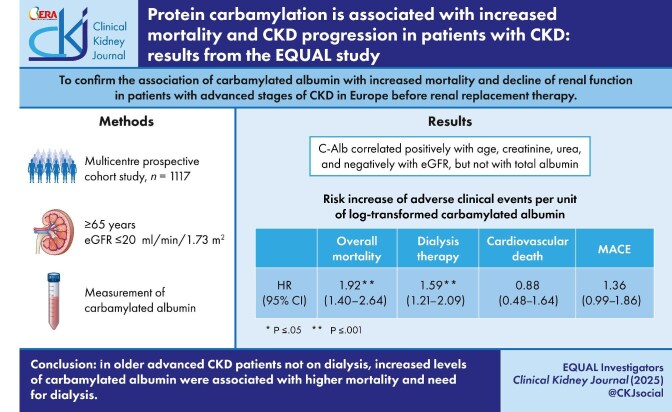

KEY LEARNING POINTS **What was known:**

- Previous studies have found that C-Alb in CKD patients was associated with increased risk for overall mortality, development of ESKD, 1-year cardiovascular mortality, and 4-year mortality from chronic heart failure.
- Even patients with healthy kidneys but high carbamylation markers had a higher risk for MACE events, heart transplantation, or death.

**This study adds:**

- So far, most studies have covered patients on dialysis or in earlier stages of CKD.
- The EQUAL cohort therefore occupies an intermediate position by looking at advanced stages of CKD before the need for dialysis, while at the same time allowing the previous results to be confirmed for European patients.

**Potential impact:**

- The role of C-Alb as an independent risk factor for mortality and progression in older patients with advanced CKD is highlighted.
- Whether reducing C-Alb results in clinical benefits for CKD patients needs to be investigated in further studies.

## INTRODUCTION

Carbamylation has been proposed as a possible pathophysiological pathway linking CKD to increased cardiovascular risk and all-cause mortality. Carbamylation is a non-enzymatic post-translational modification by irreversible covalent binding of isocyanate to the amino groups of proteins, peptides or free amino acids [1]. The primary source of isocyanate formation is the decomposition of urea [2, 3]. The ratio of carbamylated to total albumin is ∼2-fold higher in uraemic CKD patients compared to non-uraemic individuals and isocyanate concentrations reach up to 140 nmol/l in CKD patients versus about 45 nmol/l in healthy controls [4–6]. Patients in the Ludwigshafen Risk and Cardiovascular (LURIC) study with no or mild CKD showed C-Alb values between 4.7 and 6.8 mmol/mol, which were measured in the same laboratory as our results [7]. An alternative origin of protein carbamylation is thiocyanate, which can be oxidized to cyanate, reacting with proteins or amino acids and thus triggering pathologic processes [3, 8–12].

Protein modification by carbamylation chemically alters the side chains of protein lysine residues, altering their charge, size, and conformation, resulting in changes in protein function [3, 13]. A growing number of studies in CKD patients suggest adverse outcomes caused by protein carbamylation. In particular, it has been speculated that protein carbamylation contributes to uraemia-associated myocardial interstitial fibrosis and structural changes in the myocardium that may lead to hypertrophy, cardiomyopathy and subsequent adverse events [14]. Within the German Diabetes Dialysis Study (4D), baseline levels of carbamylated albumin (C-Alb) strongly correlated with 1-year cardiovascular mortality (primarily from sudden cardiac death), as well as with 4-year mortality from chronic heart failure, but not with increased risk of myocardial infarction or stroke [15]. In addition, C-Alb appears to be a potential risk marker for CKD progression. In the Chronic Renal Insufficiency Cohort study, participants in the top C-Alb quartile were more than twice as likely to develop ESKD than participants in the lowest C-Alb quartile [16].

In the present study we investigated the association between protein carbamylation and mortality, cardiovascular death, the occurrence of major adverse cardiac events (MACE) and need for dialysis therapy in older patients with advanced CKD.

## MATERIALS AND METHODS

### Study population

The EQUAL study (European Quality Study on treatment in advanced chronic kidney disease) is a multicentre international prospective cohort study initiated by the European Renal Association. In total, >1700 patients from the Netherlands, the UK, Italy, Germany, Sweden, and Poland have been included since March 2012 (Supplementary Fig. S1). Patients aged ≥65 years with an incident GFR ≤20 ml/min/1.73 m^2^ who had not previously received kidney transplantation or chronic dialysis therapy were included. If the participants’ eGFR again rose above 20 ml/min/1.73 m^2^ during the study, this would not lead to study exclusion if they otherwise fulfilled the eligibility criteria. The eGFR was calculated by using the CKD-EPI creatinine equation. Patients were followed up for a maximum of 5 years or until kidney transplantation, discontinuation of nephrology care, transfer of the patient to a non-participating centre, refusal of further participation, or death. C-Alb was measured in plasma samples obtained at the baseline visit in a subgroup of 1117 patients (Supplementary Table S1).

### Laboratory analysis

A complete description of the mass spectrometric assay for C-Alb has been previously reported [4]. C-Alb values are reported in units of mmol/mol (mmoles C-Alb per mole of uncarbamylated albumin). The intra-assay coefficient of variance for C-Alb measurements was 1.9%.

### Outcomes

The main outcome measure was all-cause mortality in patients with different C-Alb levels. In addition, the time to dialysis initiation, cardiovascular death, and the occurrence of MACE were separate endpoints. Dialysis therapy included both peritoneal dialysis and haemodialysis. MACE was defined as a comorbidity or hospitalization due to cerebrovascular disease, myocardial infarction, peripheral vascular disease, angina pectoris, arrhythmias, congestive heart failure, coronary artery disease, or death due to myocardial ischaemia and infarction, heart failure, cardiac arrest, or cerebrovascular accident. A first MACE refers to the first occurrence of a MACE after entering the study.

### Statistical analysis

For the description of the study cohort, quartiles were formed on the basis of the measured values of C-Alb at baseline. The lowest quartile served as the reference group. For the comparison of the groups, we used univariate analyses of variance and chi-square tests. Continuous variables were tested for a gaussian distribution using the Shapiro–Wilk test and reported by using means and standard deviations. Medians and interquartile ranges were described for the variables that were not normally distributed. Categorical variables were expressed in absolute numbers and percentages. Spearman rank correlation coefficients were determined to investigate the correlation between variables and C-Alb. In addition, we performed independent *t*-tests to compare mean C-Alb values across patient groups divided by the categorical variables and visualized results using box plots.

The risks of different outcomes were analysed by using Kaplan–Meier survival curves and univariable and multivariable Cox proportional hazard models. In the first multivariable Cox model, adjustment was made for the possible confounding effects of age, sex, and ethnicity. In the next step we included existing comorbidities at the time of study inclusion (diabetes, cardiovascular disease, peripheral vascular disease, chronic heart failure, cerebrovascular disease, history of myocardial infarction, as well as systolic blood pressure, BMI, and smoking status). In the following two models, laboratory parameters (albumin, haemoglobin, potassium, phosphate) and medication with ESA and ACE inhibitors, as well as the original cause of CKD were included as confounders. Further adjustment was made for eGFR and urea as possible intermediate parameters, which are potentially part of a pathophysiological pathway of protein carbamylation. In the continuous exposure models, C-Alb values were naturally log transformed. Multiple imputation was performed using five datasets with the Mersenne Twister as the random number generator. Overall, 3.6% of covariate values were missing (841/22 616 values). Competing risk analyses were performed, with death as a competing event for dialysis and non-cardiovascular death as a competing event for cardiovascular death (Supplementary Table S4). Summary statistics were reported with 95% confidence intervals and a *P* value of <.05 was considered significant. The programs IBM SPSS Statistics (v.27.0) and R (v.2025.05.1 + 513) were used for all statistical analyses.

## RESULTS

In total, 1117 patients were included, whose baseline characteristics are described in Table 1. C-Alb values ranged from 3.40to 71.34 mmol/mol with a mean of 13.5 ± 6.5 mmol/mol and a median value of 12.1 mmol/mol (Supplementary Figs S2–S6). Study participants were on average 77 ± 7 years of age, 64.0% were male, and 94.4% were white and had an average eGFR of 17.2 ± 6.6 ml/min/1.73 m^2^. Regarding comorbidities, 41.9% had been diagnosed with diabetes mellitus and 82.8% with arterial hypertension.

**Table 1:** Baseline characteristics.

	All cases	Quartile 1	Quartile 2	Quartile 3	Quartile 4
C-Alb (mmol/mol)		<9.13	9.13–12.07	12.07–15.91	>15.91
Number of patients	*n* = 1117	*n* = 279	*n* = 280	*n* = 279	*n* = 279
Age (years)	77 (7)	75 (7)	76 (7)	77 (7)	78 (7)
Sex (% male)	64.0% (715)	50.5% (141)	69.3% (194)	68.8% (192)	67.4% (188)
BMI (kg/m^2^)	28.5 (5.1)	30.4 (5.4)	28.6 (5.0)	27.9 (4.5)	27.1 (5.2)
Syst. RR (mmHg)	142 (22)	146 (21)	142 (22)	143 (21)	137 (23)
Ethnicity					
white, % (*n*)	94.4 (1055)	92.1 (257)	92.9 (260)	95.3 (266)	97.5 (272)
other, % (*n*)	5.6 (62)	7.9 (22)	7.1 (20)	4.7 (13)	2.5 (7)
Smoking status					
current smoker, % (*n*)	7.3 (81)	6.8 (19)	9.3 (26)	5.7 (16)	7.2 (20)
ex smoker, % (*n*)	42.2 (471)	41.9 (117)	42.5 (119)	42.7 (119)	41.6 (116)
non-smoker, % (*n*)	28.6 (319)	29.4 (82)	26.8 (75)	29.7 (83)	28.3 (79)
not specified, % (*n*)	21.9 (246)	21.9 (61)	21.4 (60)	21.9 (61)	22.9 (64)
Laboratory values					
eGFR (ml/min/1.73 m^2^)	17.2 [6.6]	19.8 [6.4]	17.6 [7.0]	16.1 [5.7]	15.3 [6.4]
Urea (mmol/l)	19.2 [8.8]	14.6 [4.6]	17.7 [6.8]	20.5 [6.4]	25.6 [9.6]
Creatinine (µmol/l)	289.0 (97.8)	243.0 (69.4)	278.3 (83.8)	306.7 (92.4)	328.7 (117.7)
ACR (mg/mmol)	32.2 [141.2]	31.2 [265.6]	32.9 [158.6]	39.4 [132.5]	28.2 [75.1]
Haemoglobin (mmol/l)	7.2 (0.9)	7.6 (0.9)	7.2 (0.9)	7.1 (0.9)	6.9 (0.9)
Albumin (g/l)	38.6 (5.1)	38.5 (5.6)	38.7 (5.3)	39.1 (4.6)	38.3 (4.7)
Kalium (mmol/l)	4.7 (0.6)	4.7 (0.6)	4.7 (0.6)	4.7 (0.6)	4.7 (0.6)
Calcium (mmol/l)	2.30 [0.19]	2.34 [0.16]	2.30 [0.20]	2.31 [0.18]	2.28 [0.21]
Phosphate (mmol/l)	1.3 (0.3)	1.2 (0.3)	1.2 (0.3)	1.3 (0.3)	1.4 (0.4)
Total cholesterol (mmol/l)	4.5 (1.3)	4.9 (1.5)	4.5 (1.2)	4.4 (1.2)	4.2 (1.2)
PTH (µmol/l)	15.6 [15.5]	13.9 [14.2]	15.4 [14.6]	15.5 [14.3]	18.1 [18.2]
Medication					
ESA medication, % (*n*)	22.7 (254)	6.5 (18)	18.2 (51)	31.5 (88)	34.8 (97)
ACE inhibitor, % (*n*)	2.6 (29)	1.1 (3)	2.1 (6)	3.2 (9)	3.9 (11)
Comorbidities					
Diabetes mellitus, % (*n*)	41.9 (468)	47.3 (132)	38.2 (107)	40.1 (112)	41.9 (117)
Art. hypertension, % (*n*)	82.8 (925)	82.8 (231)	79.3 (222)	83.5 (233)	85.7 (239)
Congestive heart failure, % (*n*)	16.6 (185)	13.6 (38)	12.1 (34)	16.8 (47)	23.7 (66)
Left ventricular hypertrophy, % (*n*)	20.7 (231)	11.8 (33)	14.3 (40)	24.4 (68)	32.3 (90)
Coronary artery disease, % (*n*)	26.5 (296)	23.7 (66)	27.1 (76)	30.1 (84)	25.1 (70)
Myocardial infarction, % (*n*)	17.1	14.3 (40)	21.1 (59)	16.1 (45)	16.8 (47)
	(191)				
Cardiac arrhythmia, % (*n*)	18.3 (204)	14.0 (39)	16.1 (45)	17.9 (50)	25.1 (70)
Peripheral artery disease, % (*n*)	17.9 (200)	16.1 (45)	15.4 (43)	20.4 (57)	19.7 (55)
Cerebrovascular disease, % (*n*)	13.8 (154)	14.3 (40)	13.6 (38)	14.0 (39)	13.3 (37)
Primary kidney disease					
Glomerular disease, % (*n*)	8.5 (95)	10.4 (29)	9.3 (26)	7.5 (21)	6.8 (19)
Tubulointerstitial injury, % (*n*)	8.5 (95)	10.8 (30)	10.0 (28)	7.5 (21)	5.7 (16)
Diabetes mellitus, % (*n*)	20.1 (224)	24.0 (67)	17.1 (48)	18.6 (52)	20.4 (57)
Art. hypertension, % (*n*)	34.2 (382)	31.9 (89)	34.6 (97)	36.9 (103)	33.3 (93)
Not specified, % (*n*)	28.7 (321)	22.9 (64)	29.0 (81)	29.5 (82)	33.8 (94)

**Figure 1:**
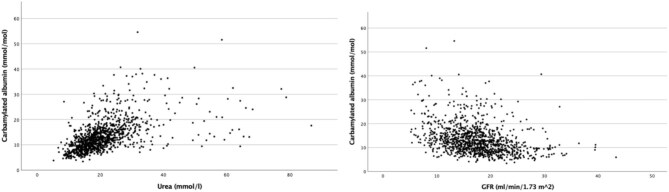
Correlations of urea and eGFR with C-Alb.

C-Alb at baseline correlated positively with age, creatinine, and urea levels. Patients with low C-Alb levels had higher eGFR and haemoglobin concentration (Fig. 1, Supplementary Table S2). There was no significant correlation with total albumin or smoking status. Men had higher C-Alb values than women, as well as patients with chronic heart failure compared to patients without (Supplementary Fig. S7, Supplementary Table S3).

**Figure 2:**
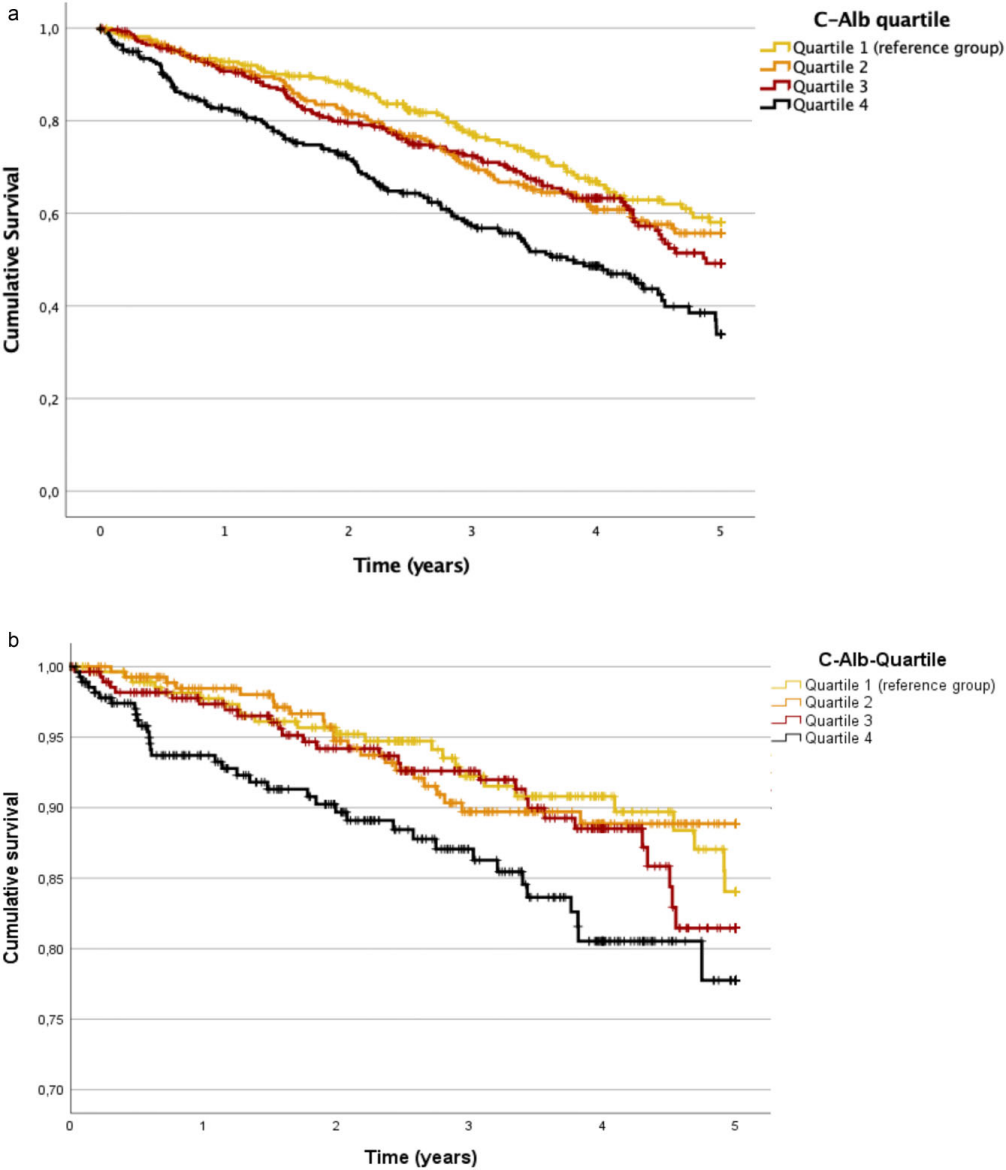

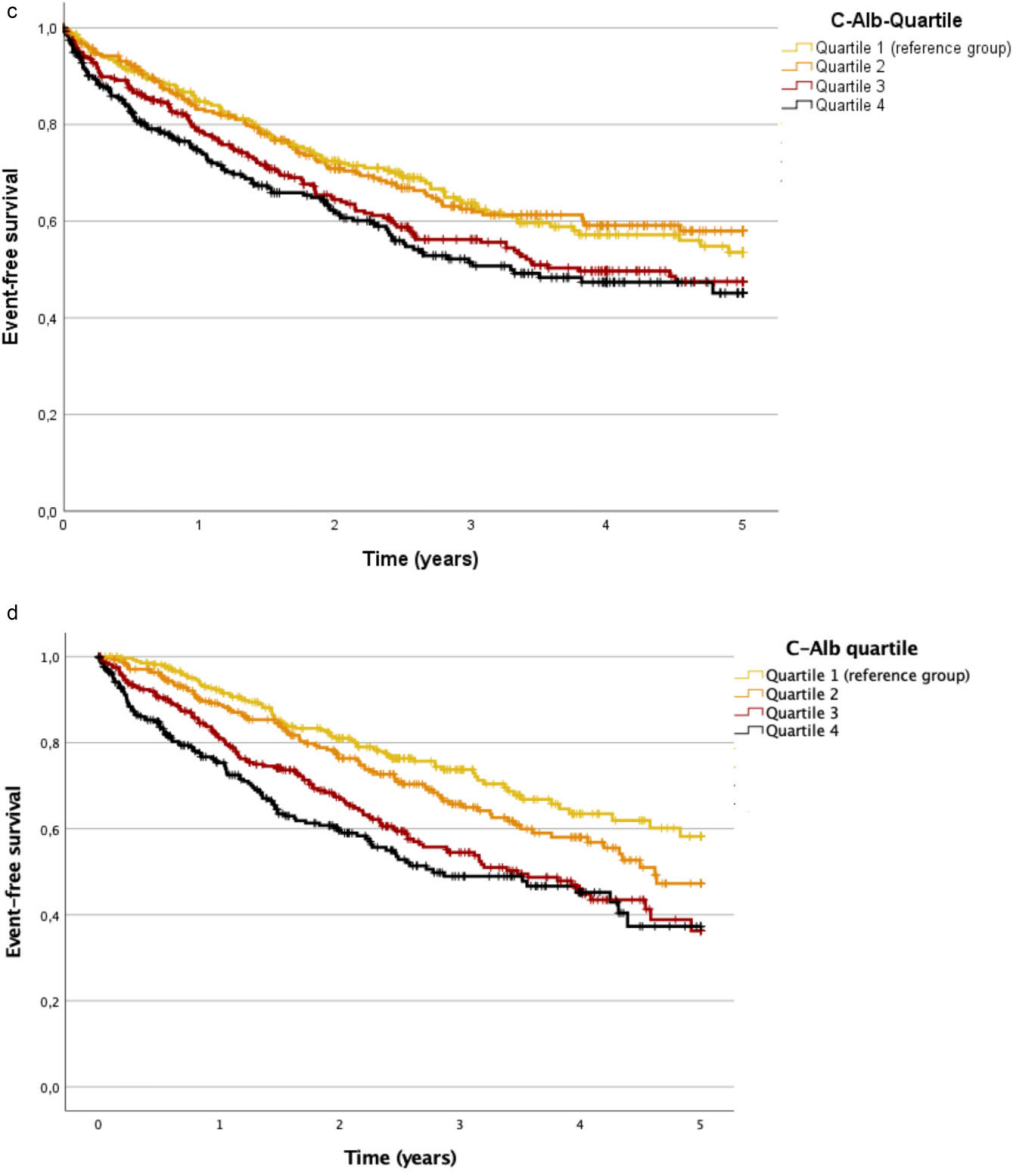
(**a**) Kaplan–Meier curves for all-cause mortality. (**b**) Kaplan–Meier curve for the outcome cardiovascular death. (**c**) Kaplan–Meier curve for the outcome MACE. (**d**) Kaplan–Meier curve for the renal outcome dialysis therapy.

During the 5 years of follow-up, 407 patients (36.4%) died and the median overall survival was 2.92 years. The Kaplan–Meier curve shows the lowest survival probability for the highest C-Alb quartile (Fig. 2a, log rank test *P* *≤* .01). Both the continuous and categorical models showed a monotonically increasing mortality risk with ascending log-transformed C-Alb concentration or C-Alb quartile (Table 2). Mortality risk increased ∼92% for each unit increase of log-transformed C-Alb (adj. HR 1.92, 95%CI 1.40–2.64, *P* ≤ .001). Compared with the lowest quartile, the highest quartile had a significantly higher mortality risk, even in the fully adjusted model (HR 1.70, 95%CI 1.20–2.39, *P* = .003).

**Table 2:** Association of C-Alb with all-cause mortality, cardiovascular death, the occurrence of MACE, and start of dialysis therapy.

	Hazard ratio (95% confidence interval)				
	Continuous Model	Quartile 1	Quartile 2	Quartile 3	Quartile 4
All-cause mortality					
Unadjusted	2.05**	Reference	1.21	1.28	2.03**
	(1.62–2.59)		(0.90–1.62)	(0.96–1.72)	(1.54–2.68)
Model 1	1.75**	Reference	1.09	1.10	1.67**
	(1.37–2.25)		(0.81–1.48)	(0.81–1.48)	(1.25–2.23)
Model 2	1.79**	Reference	1.18	1.16	1.69**
	(1.38–2.14)		(0.87–1.60)	(0.85–1.57)	(1.25–2.27)
Model 3	1.76**	Reference	1.14	1.17	1.64*
	(1.33–2.34)		(0.83–1.56)	(0.85–1.61)	(1.19–2.26)
Model 4	1.69**	Reference	1.10	1.14	1.57*
	(1.27–2.24)		(0.80–1.51)	(0.83–1.57)	(1.14–2.17)
Model 5	1.75**	Reference	1.11	1.16	1.61*
	(1.31–2.35)		(0.81–1.52)	(0.84–1.60)	(1.56–2.23)
Model 6	1.92**	Reference	1.13	1.18	1.70*
	(1.40–2.64)		(0.82–1.55)	(0.85–1.64)	(1.20–2.39)
Cardiovascular death					
Unadjusted	1.63*	Reference	0.90	1.14	1.63
	1.03–2.58		0.51–1.61	0.67–1.96	0.98–2.73
Model 1	1.49	Reference	0.88	1.08	1.47
	0.91–2.43		0.49–1.57	0.62–1.89	0.85–2.52
Model 2	1.34	Reference	0.97	1.08	1.38
	0.80–2.22		0.53–1.77	0.61–1.92	0.78–2.43
Model 3	1.16	Reference	0.94	1.02	1.20
	0.68–1.97		0.51–1.73	0.56–1.84	0.67–2.17
Model 4	1.10	Reference	0.90	0.97	1.15
	0.65–1.87		0.50–1.65	0.54–1.75	0.64–2.05
Model 5	1.06	Reference	0.90	0.94	1.11
	0.61–1.85		0.49–1.64	0.51–1.72	0.61–2.02
Model 6	0.88	Reference	0.84	0.88	0.94
	0.48–1.64		0.46–1.55	0.48–1.61	0.49–1.80
MACE					
Unadjusted	1.58**	Reference	0.97	1.31*	1.48*
	(1.25–1.98)		(0.73–1.29)	(1.00–1.70)	(1.13–1.93)
Model 1	1.43 *	Reference	0.91	1.18	1.32
	(1.13–1.81)		(0.68–1.21)	(0.90–1.54)	(1.00–1.74)
Model 2	1.43*	Reference	0.97	1.19	1.34*
	(1.11–1.83)		(0.72–1.30)	(0.90–1.57)	(1.01–1.79)
Model 3	1.33*	Reference	0.97	1.21	1.24
	(1.01–1.73)		(0.72–1.31)	(0.90–1.63)	(0.91–1.70)
Model 4	1.32*	Reference	0.97	1.21	1.23
	(1.01–1.73)		(0.72–1.31)	(0.90–1.62)	(0.90–1.68)
Model 5	1.34*	Reference	0.97	1.22	1.24
	(1.01–1.77)		(0.72–1.31)	(0.90–1.64)	(0.90–1.71)
Model 6	1.36	Reference	0.97	1.21	1.23
	(0.99–1.86)		(0.71–1.31)	(0.89–1.64)	(0.87–1.73)
Dialysis therapy					
Unadjusted	1.80**	Reference	1.29	1.61*	1.47*
	(1.43–2.28)		(0.90–1.86)	1.13–2.28	1.02–2.13
Model 1	2.12	Reference	1.33	1.80**	1.84**
	(1.66–2.70)		(0.93–1.90)	1.27–2.55	1.27–2.66
Model 2	2.36*	Reference	1.39	1.85**	1.98**
	(1.81–3.06)		(0.97–1.99)	1.29–2.64	1.35–2.90
Model 3	1.56*	Reference	1.15	1.35	1.17
	(1.19–2.04)		(0.80–1.66)	0.94–1.94	0.76–1.79
Model 4	1.59**	Reference	1.16	1.36	1.20
	(1.21– 2.09)		(0.81–1.68)	0.94–1.96	0.78–1.85

Model 1: Adjusted for age, sex, ethnicity.

Model 2: Additionally adjusted for diabetes mellitus, cardiovascular disease, peripheral vascular disease, chronic heart failure, cerebrovascular disease, myocardial infarction, systolic blood pressure, smoking status, and BMI.

Model 3: Additionally adjusted for albumin, haemoglobin, potassium, phosphate, and presence of ESA or ACE medication.

Model 4: Additionally adjusted for cause of CKD.

Model 5: Additionally adjusted for eGFR.

Model 6: Additionally adjusted for urea.

*P ≤ .05 **P ≤ .001.

In total 396 patients (35.5%) started dialysis during the study period. After 1.98 years 50% of the study population needed dialysis therapy. The Kaplan–Meier curve showed a significant difference between the two bottom and top quartiles. In continuous analysis, we found a strong association between log-transformed C-Alb at baseline and risk of dialysis initiation in the fully adjusted model (adj. HR 1.59, 95%CI 1.21–2.09, *P* ≤ .001). Across the unadjusted categorical models, the third quartile of C-Alb exhibited the highest hazard ratio for the risk of requiring dialysis therapy (HR 1.61, 95%CI 1.13–2.28, *P* = .008).

We found an association between baseline C-Alb and the risk of MACE. In the unadjusted continuous analysis, the risk of MACE increased significantly by 58% (HR 1.58, 95%CI 1.25–1.98, *P* ≤ .001) per unit of log-transformed C-Alb. Patients in the highest quartile had a 48% higher risk of MACE compared to patients in the lowest quartile in univariable analysis (HR 1.48, 95%CI 1.13–1.93, *P* = .004). These findings were attenuated in the following adjusted models.

A similar risk increase was observed for cardiovascular deaths (∼27.0% of all deaths) in the unadjusted continuous model for 63% per unit increase of log-transformed C-Alb (HR 1.63, 95%CI 1.03–2.58, *P* = .038). This association was attenuated in the adjusted models and in the categorical analysis.

## DISCUSSION

In this large European study of older adults with CKD stage 4–5, there was a strong association between higher levels of C-Alb and adverse outcomes. We observed a higher risk of both all-cause mortality and the initiation of dialysis therapy, with no significant association found between C-Alb and cardiovascular mortality or MACE.

In line with previous work suggesting that the main source C-Alb is the decomposition of urea, we observed a strong positive correlation with urea, supporting the hypothesis that prolonged uraemic conditions in CKD patients increases the carbamylation load [4, 16, 17]. A positive but less pronounced correlation with urea has also been found in studies with dialysis patients [4, 18, 19]. A possible explanation could be the decrease in protein carbamylation after the start of dialysis and the fluctuating urea levels before and after dialysis [16]. Regarding the positive correlation with age, there is evidence to suggest that carbamylation levels increase over the course of life as part of the natural ageing process [20]. As expected, there was no significant correlation of C-Alb with non-carbamylated albumin, as C-Alb was expressed as a quotient (mmol/mol), thus avoiding any influence of possible hypoalbuminaemia.

The lack of association between C-Alb levels and smoking status in the present study and MPO concentration in other studies may indicate that smoking is a minor source of cyanate, whereas uraemia is the more dominant source of carbamylation in CKD patients [16–18, 21]. Interestingly, although glycation is a possible competing post-translational modification, there was no difference in average C-Alb values between patients with or without diabetes.

To date, most clinical studies on this topic included patients on dialysis or in earlier stages of CKD [4, 15–19]. The EQUAL cohort therefore occupies an intermediate position by covering advanced stages of CKD before the need for dialysis, while at the same time allowing the previous results to be confirmed for European patients.

We observed a strong association between C-Alb and mortality risk, which is consistent with multiple clinical studies despite methodological differences [4, 8, 15–19, 22]. Even after adjustment for urea, which could also be an intermediate, the effect was still visible. One explanation for this is that (analogous to glycated haemoglobin as an indicator of time-averaged glucose concentrations), C-Alb is a better measure of time-averaged urea concentrations compared to singled urea measurements.

Second, we observed that subjects in the top two C-Alb quartiles had a higher MACE risk in the unadjusted model. Other similar studies providing evidence of the importance of carbamylation on cardiovascular risk found that patients with apparently normal renal function but high homocitrulline values also had a significantly higher risk of both future MACE events and death from MACE [8]. The LURIC study observed patients with no or mild impaired kidney function who had been referred to coronary angiography for 10 years. Higher quartiles of C-Alb were associated with an increased risk for all-cause mortality, death due to congestive heart failure, and less strongly for sudden cardiac death and fatal myocardial infarction [7].

Regarding the risk of cardiovascular death, our study suggests an increased risk in the continuous model, however, this risk was attenuated in the adjusted models probably due to the small absolute number of events (*n* = 110, 9.85% of the study population). In comparison, the French CKD-REIN study with CKD patients with comparable kidney function found a significantly increased risk for MACE and fatal and non-fatal atheromatous or nonatheromatous cardiovascular disease (554 of 2195 patients) for the top tertile of baseline homocitrulline concentration [23]. Within the German Diabetes Dialysis Study (4D), baseline levels of C-Alb were also strongly correlated with 1-year cardiovascular mortality, mainly from sudden cardiac death, and 4-year mortality from chronic heart failure, but not with increased risk of myocardial infarction or stroke [15]. And in another heart failure cohort with healthy kidney function, increasing homocitrulline levels were associated with an increased risk of heart transplantation or death in both continuous and categorical analysis [22, 24].

There are several possible mechanisms worth discussion that could mediate the proatherogenic properties of protein carbamylation.

Carbamylated proteins appear to induce direct proliferation of VSMCs and endothelial cells [25–27], increased phosphorylation of endothelial extracellular signal-regulated kinase 1, and increased cell death of endothelial cells after exposure to carbamylated LDL [26, 28]. Carbamylated LDL has also been shown to produce endothelial dysfunction, leading to the activation of NAPH oxidase via the LOX-1 receptor, uncoupling of endothelial NO synthase and increased production of reactive oxygen species (ROS) [29]. The treated endothelial cells were less responsive to acetylcholine- and calcium-mediated endothelial relaxation than nLDL-treated controls [29].

Another mechanistic link involves leukocyte accumulation, which has been shown to be increased in atherosclerotic intimal lesions and to play an important role in plaque development via subendothelial foam cell formation [24, 30]. After incubation of human coronary artery endothelial cells with cLDL, an increased expression of the leukocyte adhesion molecules ICAM-1 (intercellular adhesion molecule-1) and, to a lesser extent, VACM-1 (vascular cell adhesion molecule-1) was observed [3, 26, 31].

Protein carbamylation may also have an extracellular proatherosclerotic effect. Carbamylation causes collagen I to have local structural changes in the triple helix, to form fibrils to a lesser extent and to be degraded by collagenases to a lesser extent, preventing balanced remodelling in vessel walls [32, 33].

C-Alb seems to be a significant biomarker of global protein carbamylation, and thus the increased number of cardiovascular events and deaths observed in patients with higher C-Alb concentrations may be partially mediated by each of these mechanisms. Paradoxically, patients with high C-Alb levels and ESKD tend to have low total cholesterol levels due to protein energy wasting, low BMI and hypoalbuminaemia among other factors, and have also not benefited from statin drug therapy to reduce their cardiovascular risk in the past [15, 34]. It is therefore debated whether their increased incidence of cardiovascular events may be due to local carbamylation of LDL in atherosclerotic plaques. Another possible explanation is that, in addition to the proatherogenic effects, carbamylated tissue proteins in the heart may cause vascular calcification and stiffness, resulting in ventricular dysfunction [7, 14, 22, 35]. For instance, carbamylation has been shown to reduce the mitochondrial membrane potential in human smooth muscle cells, resulting in reduced transcription of the calcification inhibitor (ectonucleotide pyrophosphatase/phosphodiesterase 1) [36].

The risk of dialysis increased with C-Alb concentration in the continuous model, in line with previous studies [16, 37]. As in the mortality analysis, the observed effect was attenuated by confounder adjustments. These findings are consistent with experimental studies that provide a mechanistic link between carbamylation, increased renal fibrosis, and thus decline in renal function. Homocitrulline has been detected as a carbamylation marker in the basement membrane, in the mesangium, and in tubuloepithelial cells in kidney biopsies from patients with elevated urea levels, but not in kidney tissue from patients with isolated proteinuria or in freshly transplanted kidneys [38]. It has also been shown that the proteolytic activity of metalloprotease 2 (human and rat) is inhibited with increasing dose after incubation with cyanate. In previous experiments in which metalloprotease 2 was damaged by drugs, this also led to increased glomerulosclerosis [38]. In another *in vitro* experiment in glomerular mesangial cells, which were incubated with carbamylated bovine plasma proteins, an accumulation of collagen I and IV was observed compared to controls without a parallel increase in total protein biosynthesis or increased collagen degradation [39]. *In vivo*, selective fibrotic changes were observed in the tubuloepithelial cells of open Axolotl nephrons in contact with the C-Alb after 10 days [40].

Our results show that the risk of kidney replacement therapy is highest in the third quartile and then decreases. This could be explained by our competing event analysis, which takes into account deaths before dialysis therapy begins. The CKD-REIN study also observed an increased mortality risk with higher carbamylation burden before receiving kidney replacement therapy, suggesting patients in the fourth quartile may die before ESKD requiring dialysis [23].

In addition to potentially nephrotoxic and mortality-increasing pathomechanisms, numerous other potential effects of carbamylation have been described, such as on the efficacy of EPO therapy [41], haemostasis [42–44], immunological processes [33, 45], and possible malnutrition [46, 47].

It is important to consider the limitations of this study. First, our multinational European study population is much less ethnically diverse than comparable American studies, so our conclusions are only applicable to the Caucasian population. Second, we observed a small number of cardiovascular deaths, which resulted in a relatively low statistical power for this endpoint. The number of heart failure events in our dataset was too low to allow for a reliable analysis. Therefore, we were unable to examine heart failure as an endpoint, which may limit comparability with previous studies that reported on this outcome. Despite the large number of confounding variables representing relevant prognostic factors for the selected endpoints, there may be other unknown factors leading to residual confounding.

Another limitation is the lack of longitudinal C-Alb measurements. With this information, it would have been possible to investigate mortality risks associated with the C-Alb slope or changes of C-Alb after dialysis initiation [19]. Finally, due to the observational study design, it was not possible to attribute causality to our results.

The fact that higher C-Alb values were associated with both CKD progression and mortality means that patients with the highest C-Alb values required dialysis sooner and expired sooner than other CKD stage 4–5 patients who otherwise had equivalent measures of kidney function. Although this cohort of patients did not meet strict criteria for initiation of kidney replacement therapy at the time of enrolment, these studies findings provide intriguing evidence suggesting that C-Alb may be a useful biomarker for predicting which patients may benefit from early initiation of dialysis. Future controlled trials are needed to explore this clinical application of C-Alb measurements.

Future research could investigate potential interventions to reduce protein carbamylation. For instance, amino acid supplementation may serve as an alternative substrate for carbamylation reactions and thereby reduce protein carbamylation [48]. Intensified dialysis therapy has been shown to lower C-Alb levels in the ARMORR trial, and dietary strategies aimed at reducing urea load may represent another potential approach [19, 47]. These strategies should be regarded as hypotheses that warrant formal testing in appropriately designed clinical studies.

In conclusion, C-Alb is associated with an increased risk for mortality and disease progression in older adults with advanced CKD. Whether reducing C-Alb results in clinical benefits for CKD patients needs to be investigated in further studies.

## Supplementary Material

sfaf302_Supplemental_Files

## Data Availability

Data will be available upon request.
