# Supplementary material for: Protein carbamylation is associated with increased mortality and CKD progression in patients with CKD: results from the EQUAL study
Source: Clin Kidney J. 2025 Oct 15;18(11):sfaf302. doi: 10.1093/ckj/sfaf302 (PMC12585524; doi:10.1093/ckj/sfaf302)
Supplement: sfaf302_Supplemental_Files [file sfaf302_supplemental_files.zip › 435 Supplemental material.docx]

**Supplemental material:**

Figure 1: The EQUAL collective stratified by nationality

EQUAL participants

n = 1729

C-Alb not measured

n = 612

C-Alb available

n = 1117

UK

n = 420

Italy

n = 291

Netherlands

n = 208

Germany

n = 130

Poland

n = 68

Figure 2: Histogramm of the overall distribution of carbamylated albumin


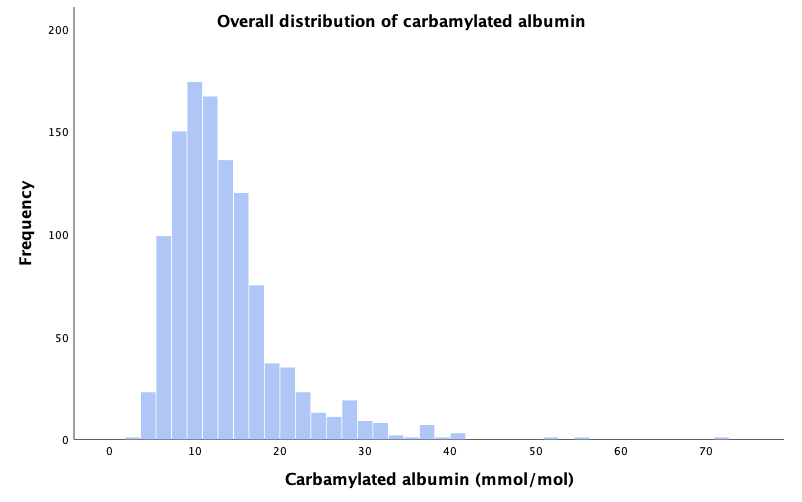


Figure 3: Histogramm of the distribution of carbamylated albumin in quartile 1


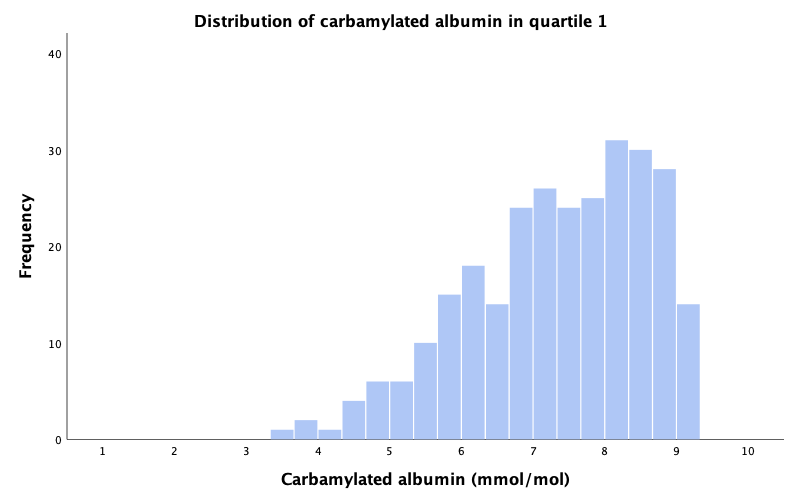


Figure 4: Histogram of the distribution of carbamylated albumin in quartile 2


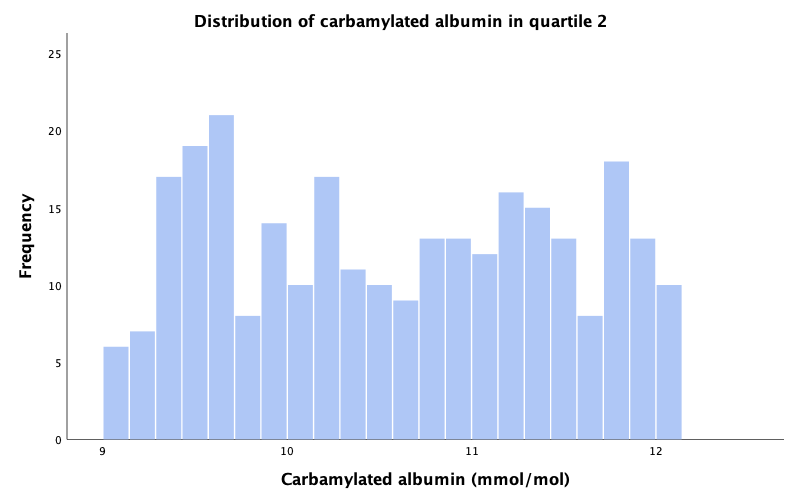


Figure 5: Histogramm of the distribution of carbamylated albumin in quartile 3


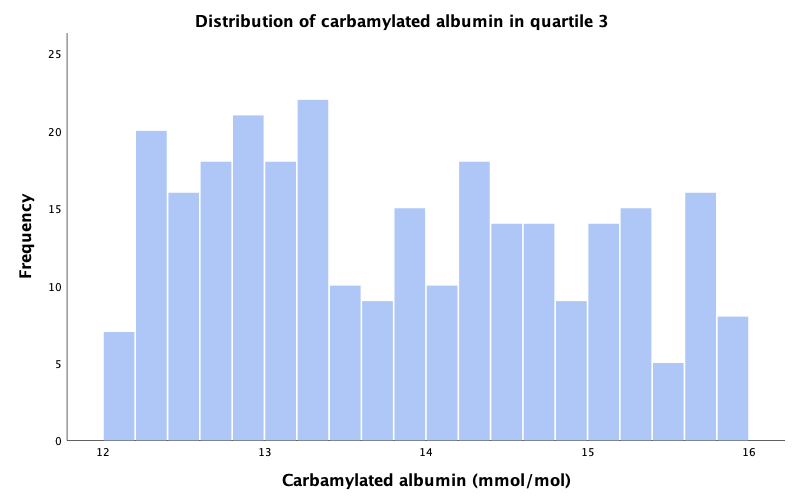


Figure 6: Histogramm of the distribution of carbamylated albumin in quartile 4


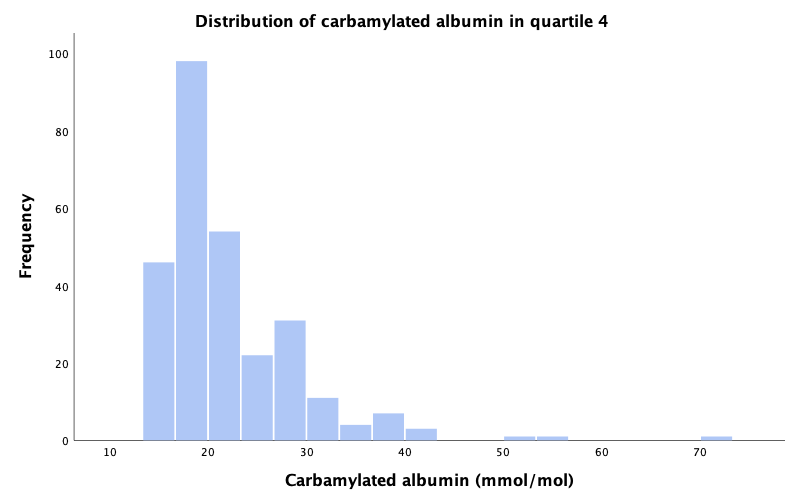


Figure 7: Box plots for mean comparisons of C-Alb stratified by gender, presence of chronic heart failure, left ventricular hypertrophy or ESA medication. For presentability, three C-Alb values above 40mmol/mol were masked out in each figure.


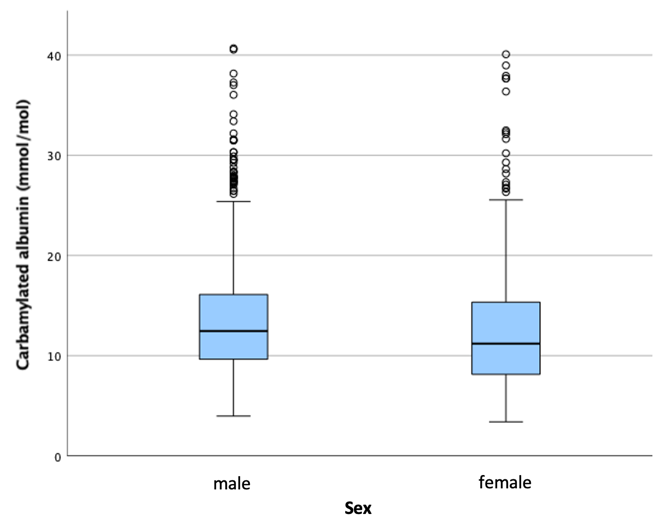

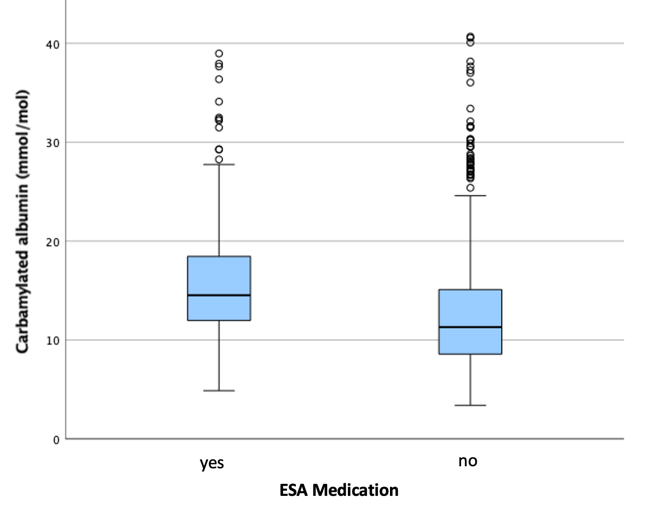


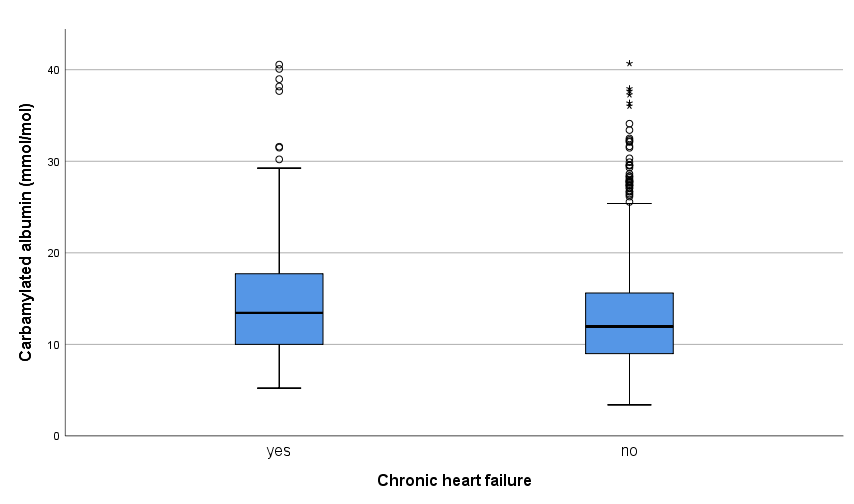

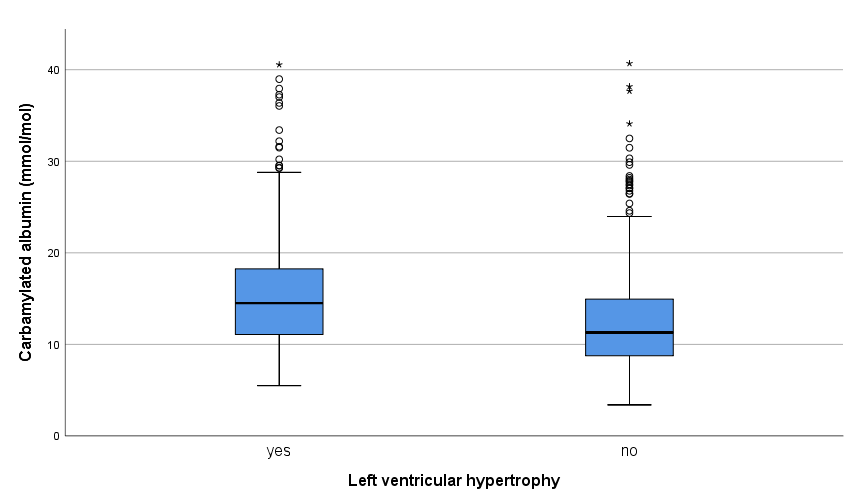


Table 1: Baseline characteristics of all EQUAL and cases with C-Alb measurements

|  |  | **Valid cases n=** | **All EQUAL cases** | **All cases with C_Alb Sample** |
| --- | --- | --- | --- | --- |
| Number of patients | |  | n = 1731 | n = 1117 |
| Age (years) | | 1731 | 76 (7) | 77 (7) |
| Sex (% male) | | 1731 | 65.5% (1133) | 64.0% (715) |
| BMI (kg/m^2^) | | 1576 | 28.4 (5.3) | 28.5 (5.1) |
| Syst. RR (mmHG) | | 1676 | 143 (22) | 142 (22) |
| **Ethnicity** | white | 1731 | 95.0% (1644) | 94.4% (1055) |
|  | other |  | 5.0% (87) | 5.6% (62) |
| **Smoking status** | current smoker | 1731 | 7.0% (121) | 7.3% (81) |
|  | ex smoker |  | 43.2% (748) | 42.2% (471) |
|  | non smoker |  | 29.7% (514) | 28.6% (319) |
|  | not specified |  | 20.1% (348) | 21.9% (246) |
| **Labaratory values** | eGFR (ml/min/1.73m^2) | 1698 | 17.0 [6.2] | 17.2 [6.6] |
|  | Urea (mmol/l) | 1638 | 19.3 [8.9] | 19.2 [8.8] |
|  | Creatinine (µmol/l) | 1698 | 292.4 (97.8) | 289.0 (97.8) |
|  | ACR (mg/mmol) | 761 | 34.1 [148.5] | 32.2 [141.2] |
|  | Hemoglobin (mmol/l) | 1680 | 7.2 (0.9) | 7.2 (0.9) |
|  | Albumin (g/l) | 1537 | 38.0 (5.1) | 38.6 (5.1) |
|  | Kalium (mmol/l) | 1692 | 4.6 (0.6) | 4.7 (0.6) |
|  | Calcium (mmol/l) | 1632 | 2.30 [0.19] | 2.30 [0.19] |
|  | Phosphate (mmol/l) | 1607 | 1.3 (0.3) | 1.3 (0.3) |
|  | Total cholesterol (mmol/l) | 1285 | 4.6 (1.3) | 4.5 (1.3) |
|  | PTH (µmol/l) | 1382 | 15.4 [14.7] | 15.6 [15.5] |
| **Medication** | ESA medication | 1709 | 24.5% (424) | 22.7% (254) |
|  | ACE inhibitor | 1696 | 2.7% (46) | 2.6% (29) |
| **Comorbidities** | Diabetes mellitus (%) | 1677 | 40.8% (706) | 41.9% (468) |
|  | Art. Hypertension | 1637 | 84.2% (1457) | 82.8% (925) |
|  | Congestive heart failure | 1632 | 17.2% (298) | 16.6% (185) |
|  | Left ventricular hypertrophy | 1501 | 21.1% (365) | 20.7% (231) |
|  | Coronary artery disease | 1643 | 26.0% (450) | 26.5% (296) |
|  | Myocardial infarction | 1680 | 17.0% (294) | 17.1% (191) |
|  | Cardiac arrhytmia | 1661 | 17.7% (306) | 18.3% (204) |
|  | Peripheral artery disease | 1654 | 16.6% (287) | 17.9% (200) |
|  | Cerebrovasc. disease | 1668 | 14.8% (256) | 13.8% (154) |
| **Primary kidney disease** | Glomerular disease | 1731 | 9.1% (158) | 8.5% (95) |
|  | Tubulointerstitial injury |  | 8.4% (146) | 8.5% (95) |
|  | Diabetes mellitus |  | 20.1% (348) | 20.1% (224) |
|  | Art. Hypertension |  | 35.3% (611) | 34.2% (382) |
|  | not specified |  | 27.1% (468) | 28.7% (321) |

Table 2: Univariable analyses of variance (ANOVA) and chi-square tests of continuous variables with carbamylated albumin

| **Correlations** | **r=** | **p=** |
| --- | --- | --- |
| Urea | 0,620 | < 0,001 |
| Creatinine | 0,352 | < 0,001 |
| Phosphate | 0,315 | < 0,001 |
| Age | 0,169 | < 0,001 |
| PTH | 0,117 | < 0,001 |
| ACR | - 0,120 | 0,020 |
| Systolic blood pressure | - 0,135 | < 0,001 |
| Calcium | - 0,157 | < 0,001 |
| Total cholesterol | - 0,159 | < 0,001 |
| BMI | - 0,259 | < 0,001 |
| Hemoglobin | - 0,295 | < 0,001 |
| eGFR | - 0,381 | < 0,001 |
| Potassium | 0,035 | 0,254 |
| Albumin | - 0,049 | 0,130 |

Table 3: Mean comparisons of categorical variables with carbamylated albumin

| **Mean comparisons** |  | **Mean ± SD** | **p=** |
| --- | --- | --- | --- |
| Sex | Male | 13,87 ± 6,61 | 0,003 |
|  | Female | 12,67 ± 6,29 |  |
| Smoking status | Smoker | 12,86 ± 5,57 | 0,725 |
|  | Ex-smoker | 13,31 ± 6,42 |  |
|  | Non-smoker | 13,55 ± 6,25 |  |
| Diabetes mellitus | yes | 13,15 ± 6,04 | 0,155 |
|  | no | 13,72 ± 6,93 |  |
| Arterial Hypertension | yes | 13,54 ± 6,57 | 0,349 |
|  | no | 12,97 ± 6,67 |  |
| ESA Medication | yes | 16,11 ± 6,99 | < 0,001 |
|  | no | 12,64 ± 6,19 |  |
| Chronic heart failure | yes | 15,42 ± 8,61 | < 0,001 |
|  | no | 13,05 ± 5,97 |  |
| CAD | yes | 13,62 ± 6,91 | 0,555 |
|  | no | 13,35 ± 6,35 |  |
| PAD | yes | 14,13 ± 6,77 | 0,113 |
|  | no | 13,31 ± 6,52 |  |
| Left ventricular hypertrophy | yes | 15,93 ± 7,27 | < 0,001 |
|  | no | 12,53 ± 6,04 |  |
| Cardiac arrhythmia | yes | 14,87 ± 7,27 | 0,001 |
|  | no | 13,14 ± 6,33 |  |
| Cerebrovascular disease | yes | 13,47 ± 7,01 | 0,997 |
|  | no | 13,47 ± 6,47 |  |

Table 4: Association of carbamylated albumin with all-cause mortality, cardiovascular death, the occurrence of MACE and start of dialysis therapy (using Baseline data without Multiple Imputation or competing event analysis)

|  |  | | **Hazard Ratio (95% Confidence Interval)** | | | | |
| --- | --- | --- | --- | --- | --- | --- | --- |
|  | **Events** | | **Continuous Model** | **Quartile 1** | **Quartile 2** | **Quartile 3** | **Quartile 4** |
| **All-cause mortality** | | | | | | | |
| Unadjusted | n = 1117 events = 407 | | 2,053 ** (1,624 – 2,594) | Reference | 1,207 (0,898 – 1,624) | 1,284 (0,959 - 1719) | 2,029 ** (1,536 – 2,680) |
| Model 1 | n= 1117 events = 407 | | 1,753 ** (1,368 – 2,246) | Reference | 1,094 (0,809 – 1,479) | 1,097 (0,813 – 1,480) | 1,668 ** (1,250 – 2,226) |
| Model 2 | n = 902 events = 328 | | 1,811 ** (1,356 – 2,418) | Reference | 1,232 (0,868 – 1,749) | 1,238 (0,880 – 1,743) | 1,760 ** (1,256 – 2,466) |
| Model 3 | n = 781 events = 289 | | 1,710 * (1,217 – 2,403) | Reference | 1,075 (0,734 – 1,575) | 1,179 (0,804 – 1,730) | 1,584 * (1,077 – 2,330) |
| Model 4 | n = 780 events = 289 | | 1,633 * (1,166 – 2,287) | Reference | 1,056 (0,720 – 1,547) | 1,155 (0,788 – 1,693) | 1,530 * (1,040 – 2,251) |
| Model 5 | n = 780 events = 289 | | 1,676 * (1,186 – 2,368) | Reference | 1,061 (0,723 – 0,556) | 1,169 (0,794 – 1,722) | 1,552 * (1,048 – 2,299) |
| Model 6 | n= 766 events = 282 | | 1,818 * (1,235 – 2,675) | Reference | 1,113 (0,752 – 1,648) | 1,227 (0,825 – 1,825) | 1,696 * (1,111 – 2,589) |
| **Cardiovascular death** | | | | |  |  |  |
| Unadjusted | n = 1110 events = 110 | | 1,985 * (1,264 – 3,115) | Reference | 0,945 (0,530 – 1,685) | 1,198 (0,694 – 2,068) | 1,906 * (1,136 – 3,198) |
| Model 1 | n= 1110 events = 110 | | 1,721 * (1,075 – 2,755) | Reference | 0,877 (0,486 – 1,581) | 1,063 (0,608 – 1,856) | 1,606 (0,941 – 2,739) |
| Model 2 | n = 895 events = 89 | | 1,357 (0,786 – 2,342) | Reference | 1,139 (0,579 – 2,242) | 1,332 (0,710 – 2,499) | 1,484 (0,784 – 2,811) |
| Model 3 | n = 774 events = 77 | | 0,958  (0,501 – 1,832) | Reference | 1,046 (0,498 – 2,194) | 1,250 (0,605 – 2,582) | 1,171 (0,551 – 2,489) |
| Model 4 | n = 773 events = 77 | | 0,927 (0,492 – 1,748) | Reference | 1,045 (0,496 – 2,199) | 1,266 (0,613 – 2,615) | 1,113 (0,524 – 2,364) |
| Model 5 | n = 773 events = 77 | | 0,955 (0,500 – 1,824) | Reference | 1,050 (0,499 – 2,206) | 1,303 (0,628 – 2,704) | 1,148 (0,538 – 2,448) |
| Model 6 | n= 759 events = 72 | | 0,709  (0,348 – 1,445) | Reference | 0,946 (0,431 – 2,075) | 1,240 (0,579 – 2,657) | 0,953 (0,418 – 2,172) |
| **MACE** | | | | | | | |
| Unadjusted | n = 1117  events = 428 | | 1,576 ** (1,253 – 1,983) | Reference | 0,973 (0,733 – 1,290) | 1,305 * (1,000 – 1,703) | 1,477 * (1,129 – 1,933) |
| Model 1 | n= 1117 events = 428 | | 1,428 * (1,125 – 1,813) | Reference | 0,911 (0,683 – 1,213) | 1,176 (0,895 – 1,544) | 1,318 (0,999 – 1,738) |
| Model 2 | n = 902 events = 344 | | 1,444 *  (1,096 – 1,903) | Reference | 1,210 (0,868 – 1,686) | 1,371 (0,999 – 1,881) | 1,467 * (1,056 – 2,039) |
| Model 3 | n = 781 events = 299 | | 1,378 * (1,000 – 1,899) | Reference | 1,337 (0,926 – 1,931) | 1,550 * (1,073 – 2,239) | 1,444 (0,983 – 2,121) |
| Model 4 | n = 780 events = 299 | | 1,342 (0,973 – 1,851) | Reference | 1,304 (0,904 – 1,882) | 1,491 * (1,031 – 2,158) | 1,372 (0,933 – 2,016) |
| Model 5 | n = 780 events = 299 | | 1,374 (0,989 – 1,909) | Reference | 1,311 (0,909 – 1,893) | 1,514 * (1,043 – 2,198) | 1,400 (0,947 – 2,070) |
| Model 6 | n= 766 events = 290 | | 1,376  (0,950 – 1,993) | Reference | 1,338 (0,915 – 1,957) | 1,529 * (1,040 – 2,248) | 1,410 (0,926 – 2,148) |
| **Dialysis therapy** | | | | | | | |
| Unadjusted | n = 1117 events = 396 | | 2,390 ** (1,876 – 3,044) | Reference | 1,310 (0,962 – 1,784) | 1,920 ** (1,434 – 2,570) | 2,335 ** (1,739 – 3,314) |
| Model 1 | n = 1117 events = 396 | | 2,826 ** (2,203 – 3,636) | Reference | 1,309 (0,957 – 1,789) | 2,044 ** (1,519 – 2,750) | 2,693 ** (1,990 – 3,644) |
| Model 2 | n = 902 events = 325 | | 3,145 ** (2,349 – 4,210) | Reference | 1,480 * (1,043 – 2,101) | 1,948 ** (1,388 – 2,734) | 3,014 ** (2,137 – 4,252) |
| Model 3 | n =781 events = 280 | | 1,915 ** (1,367 – 2,682) | Reference | 1,111 (0,756 – 0,1633) | 1,207 (0,817 – 1,782) | 1,730 * (1,165 – 2,569) |
| Model 4 | n = 780 events = 280 | | 1,904 ** (1,345 – 2,677) | Reference | 0,622 (0,750 – 1,618) | 1,192 (0,807 – 1,760) | 1,686 * (1,135 – 2,505) |
|  | | Model 1: Adjusted for age, sex, ethnitcity  Model 2: Additionally adjusted for diabetes mellitus, cardiovascular disease, perioheral vascular disease, chronic heart failure, cerebrovascular disease, myocardial infactation, systolic blood pressure, smoking status and BMI  Model 3: Additionally adjusted for albumin, hemoglobin, potassium, phosphate and presence of ESA oder ACE medication  Model 4: Additionally adjusted for cause of CKD  Model 5: Additionally adjusted for eGFR  Model 6: Additionally adjusted for urea  * p= <0,05 ** p=<0,001 | | | | | |
