# Supplementary material for: Protein carbamylation is associated with increased mortality and CKD progression in patients with CKD: results from the EQUAL study
Source: Clin Kidney J. 2025 Oct 15;18(11):sfaf302. doi: 10.1093/ckj/sfaf302 (PMC12585524; doi:10.1093/ckj/sfaf302)
Supplement: sfaf302_Supplemental_Files [file sfaf302_supplemental_files.zip › Supplemental material Revision.pdf]

Supplemental material:

Figure 1: The EQUAL collective stratified by nationality

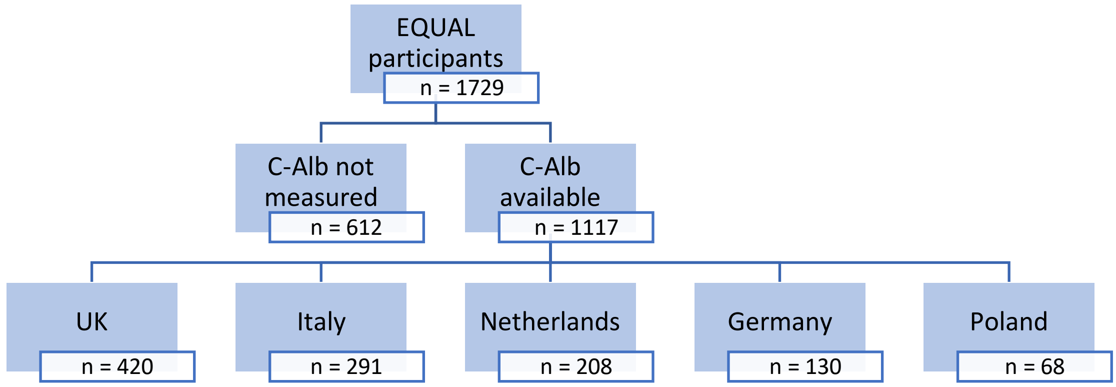

Figure 2: Histogramm of the overall distribution of carbamylated albumin

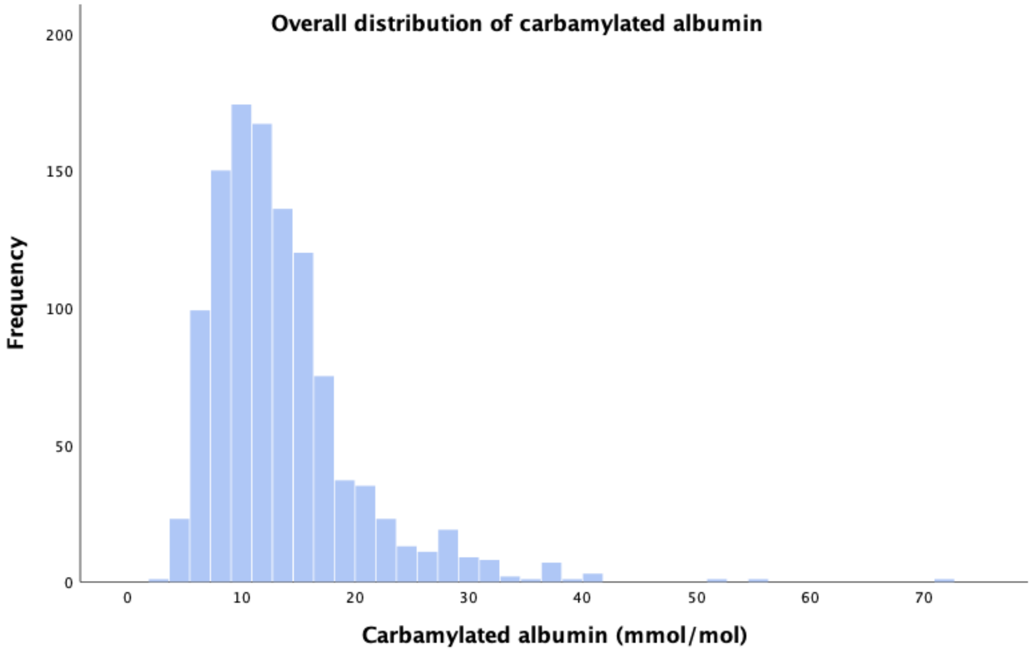

Figure 3: Histogramm of the distribution of carbamylated albumin in quartile 1

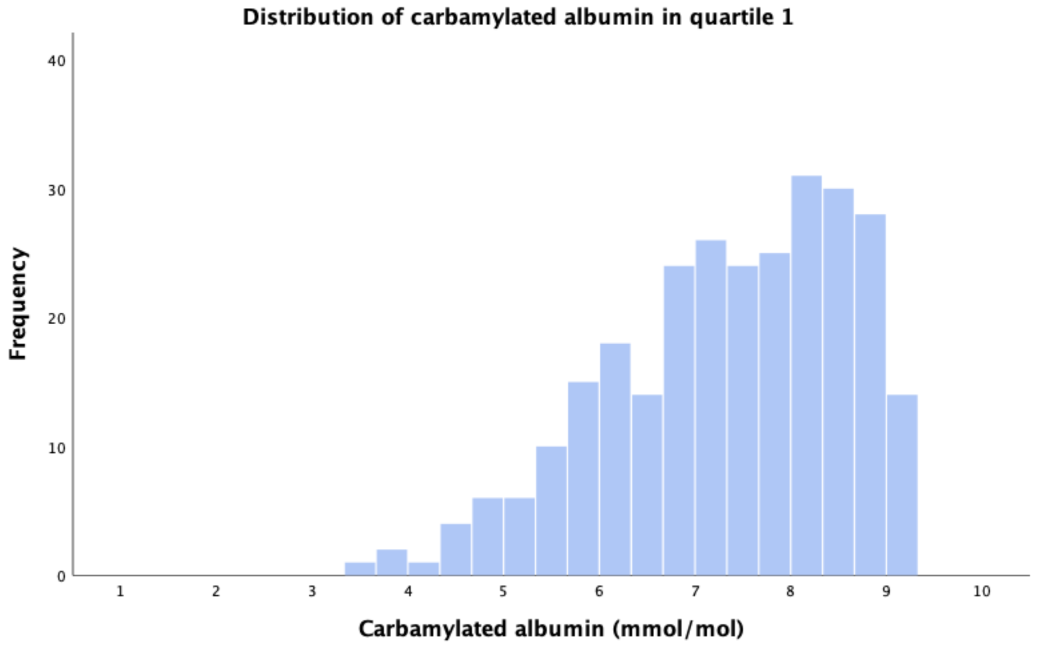

Figure 4: Histogramm of the distribution of carbamylated albumin in quartile 2

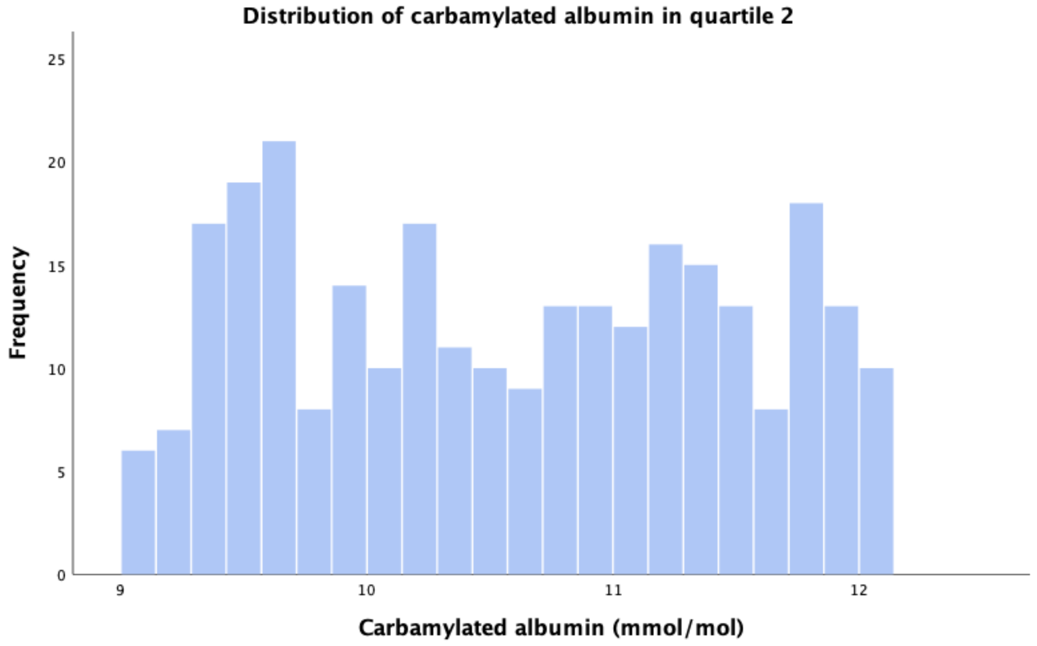

Figure 5: Histogramm of the distribution of carbamylated albumin in quartile 3

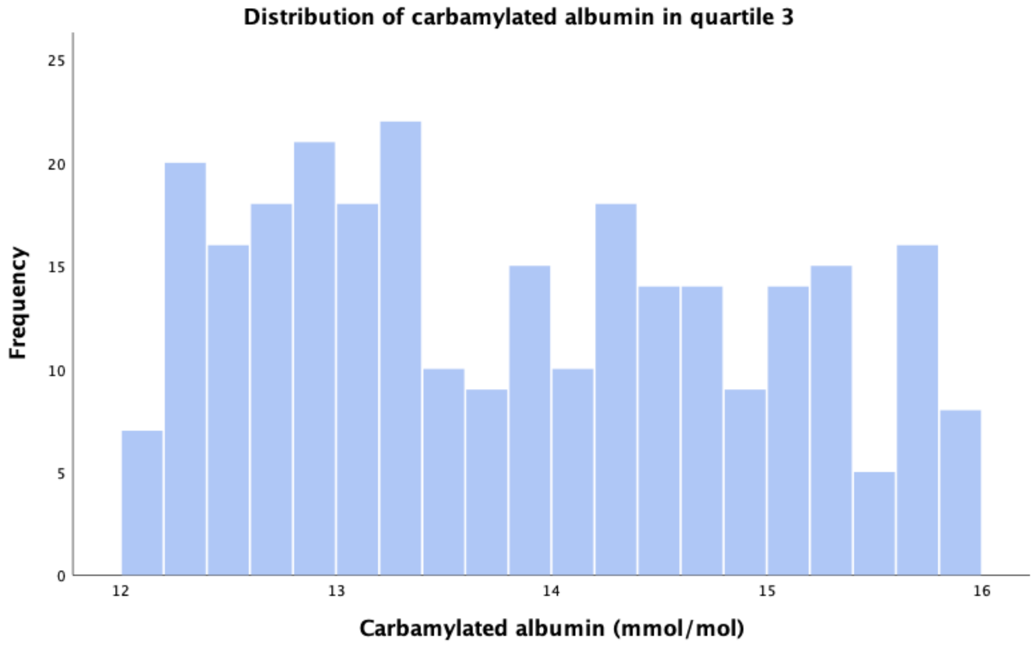

Figure 6: Histogramm of the distribution of carbamylated albumin in quartile 4

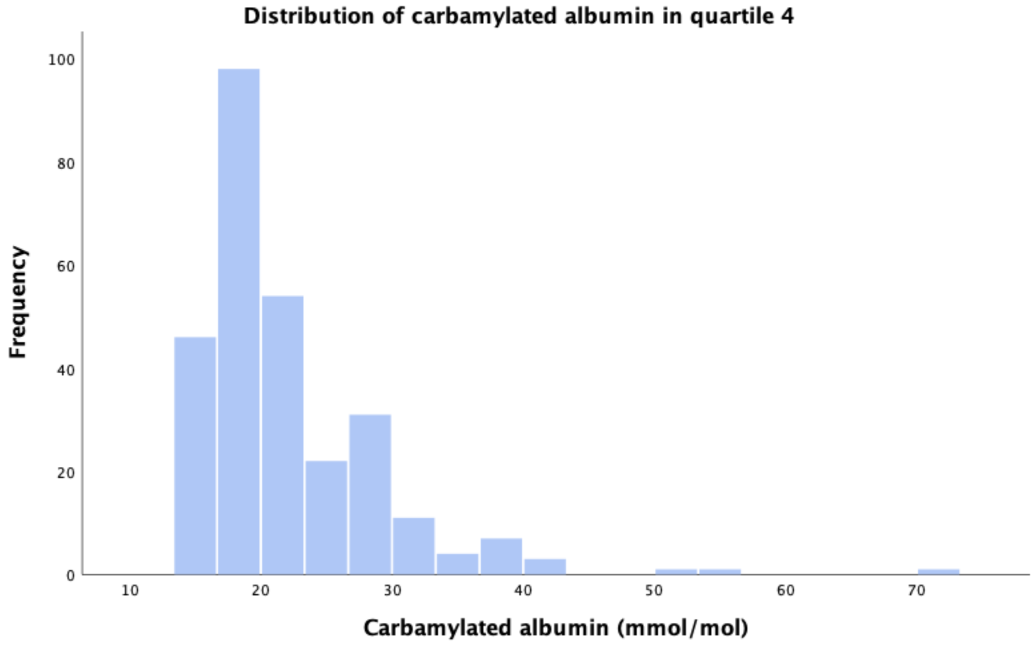

Figure 7: Box plots for mean comparisons of C-Alb stratified by gender, presence of chronic heart failure, left ventricular hypertrophy or ESA medication. For presentability, three C-Alb values above 40mmol/mol were masked out in each figure.

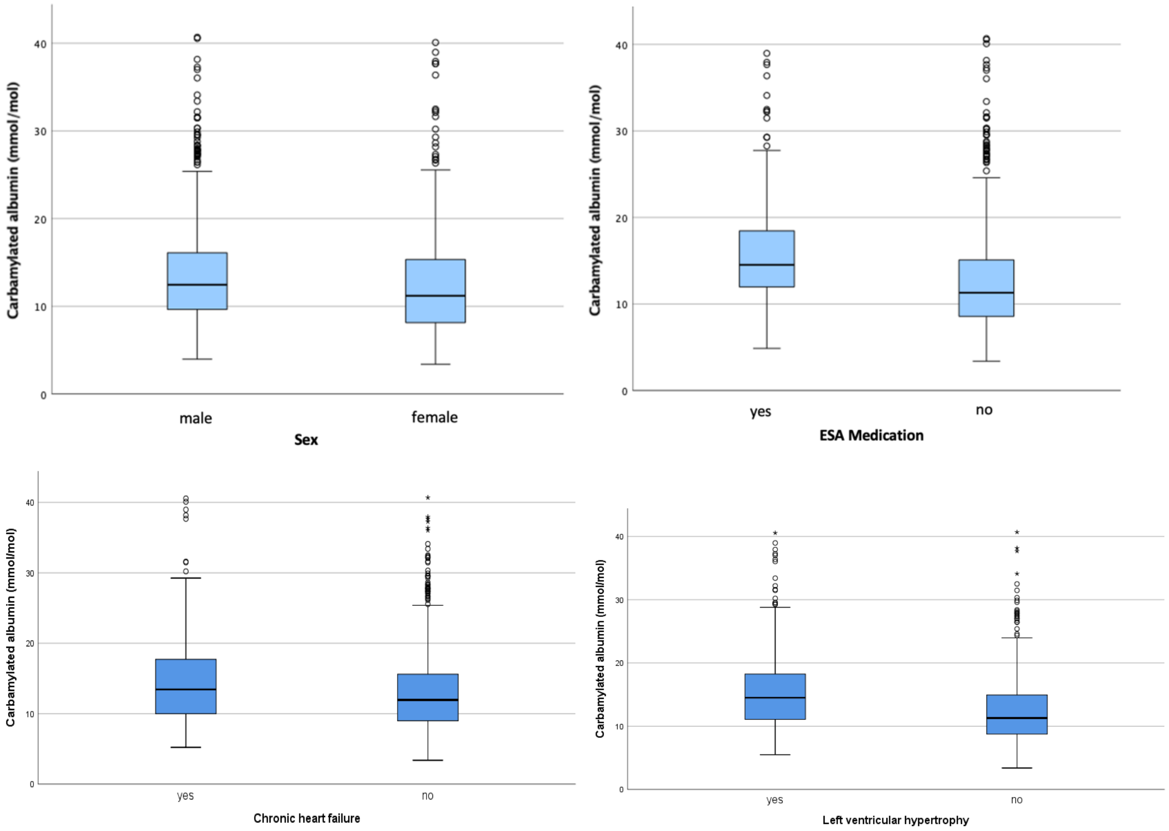

Table 1: Baseline characteristics of all EQUAL and cases with C-Alb measurements

		Valid cases n=	All EQUAL cases	All cases with C_Alb Sample
Number of patients			n = 1731	n = 1117
Age (years)		1731	76 (7)	77 (7)
Sex (% male)		1731	65.5% (1133)	64.0% (715)
BMI (kg/m^2)		1576	28.4 (5.3)	28.5 (5.1)
Syst. RR (mmHG)		1676	143 (22)	142 (22)
**Ethnicity**	white	1731	95.0% (1644)	94.4% (1055)
	other		5.0% (87)	5.6% (62)
**Smoking status**	current smoker	1731	7.0% (121)	7.3% (81)
	ex smoker		43.2% (748)	42.2% (471)
	non smoker		29.7% (514)	28.6% (319)
	not specified		20.1% (348)	21.9% (246)
**Labaratory values**	eGFR (ml/min/1.73m^2)	1698	17.0 [6.2]	17.2 [6.6]
	Urea (mmol/l)	1638	19.3 [8.9]	19.2 [8.8]
	Creatinine (µmol/l)	1698	292.4 (97.8)	289.0 (97.8)
	ACR (mg/mmol)	761	34.1 [148.5]	32.2 [141.2]
	Hemoglobin (mmol/l)	1680	7.2 (0.9)	7.2 (0.9)
	Albumin (g/l)	1537	38.0 (5.1)	38.6 (5.1)
	Kalium (mmol/l)	1692	4.6 (0.6)	4.7 (0.6)
	Calcium (mmol/l)	1632	2.30 [0.19]	2.30 [0.19]
	Phosphate (mmol/l)	1607	1.3 (0.3)	1.3 (0.3)
	Total cholesterol (mmol/l)	1285	4.6 (1.3)	4.5 (1.3)
	PTH (µmol/l)	1382	15.4 [14.7]	15.6 [15.5]
**Medication**	ESA medication	1709	24.5% (424)	22.7% (254)
	ACE inhibitor	1696	2.7% (46)	2.6% (29)
**Comorbidities**	Diabetes mellitus (%)	1677	40.8% (706)	41.9% (468)
	Art. Hypertension	1637	84.2% (1457)	82.8% (925)
	Congestive heart failure	1632	17.2% (298)	16.6% (185)
	Left ventricular hypertrophy	1501	21.1% (365)	20.7% (231)
	Coronary artery disease	1643	26.0% (450)	26.5% (296)
	Myocardial infarction	1680	17.0% (294)	17.1% (191)
	Cardiac arrhytmia	1661	17.7% (306)	18.3% (204)
	Peripheral artery disease	1654	16.6% (287)	17.9% (200)
	Cerebrovasc. disease	1668	14.8% (256)	13.8% (154)
**Primary kidney disease**	Glomerular disease	1731	9.1% (158)	8.5% (95)
	Tubulointerstitial injury		8.4% (146)	8.5% (95)
	Diabetes mellitus		20.1% (348)	20.1% (224)
	Art. Hypertension		35.3% (611)	34.2% (382)
	not specified		27.1% (468)	28.7% (321)

Table 2: Univariable analyses of variance (ANOVA) and chi-square tests of continuous variables with carbamylated albumin

**Correlations**	**r=**	**p=**
Urea	0,620	< 0,001
Creatinine	0,352	< 0,001
Phosphate	0,315	< 0,001
Age	0,169	< 0,001
PTH	0,117	< 0,001
ACR	- 0,120	0,020
Systolic blood pressure	- 0,135	< 0,001
Calcium	- 0,157	< 0,001
Total cholesterol	- 0,159	< 0,001
BMI	- 0,259	< 0,001
Hemoglobin	- 0,295	< 0,001
eGFR	- 0,381	< 0,001
Potassium	0,035	0,254
Albumin	- 0,049	0,130

Table 3: Mean comparisons of categorical variables with carbamylated albumin

**Mean comparisons**		**Mean ± SD**	**p=**
Sex	Male	13,87 ± 6,61	0,003
	Female	12,67 ± 6,29	
Smoking status	Smoker	12,86 ± 5,57	0,725
	Ex-smoker	13,31 ± 6,42	
	Non-smoker	13,55 ± 6,25	
Diabetes mellitus	yes	13,15 ± 6,04	0,155
	no	13,72 ± 6,93	
Arterial Hypertension	yes	13,54 ± 6,57	0,349
	no	12,97 ± 6,67	
ESA Medication	yes	16,11 ± 6,99	< 0,001
	no	12,64 ± 6,19	
Chronic heart failure	yes	15,42 ± 8,61	< 0,001
	no	13,05 ± 5,97	
CAD	yes	13,62 ± 6,91	0,555
	no	13,35 ± 6,35	
PAD	yes	14,13 ± 6,77	0,113
	no	13,31 ± 6,52	
Left ventricular hypertrophy	yes	15,93 ± 7,27	< 0,001
	no	12,53 ± 6,04	
Cardiac arrhythmia	yes	14,87 ± 7,27	0,001
	no	13,14 ± 6,33	
Cerebrovascular disease	yes	13,47 ± 7,01	0,997
	no	13,47 ± 6,47	

Table 4: Association of carbamylated albumin with all-cause mortality, cardiovascular death, the occurrence of MACE and start of dialysis therapy (using Baseline data without Multiple Imputation or competing event analysis)

		Hazard Ratio (95% Confidence Interval)				
	Events	Continuous Model	Quartile 1	Quartile 2	Quartile 3	Quartile 4
**All-cause mortality**						
Unadjusted	n = 1117<br>events = 407	2,053 **<br>(1,624 – 2,594)	Reference	1,207<br>(0,898 – 1,624)	1,284<br>(0,959 - 1719)	2,029 **<br>(1,536 – 2,680)
Model 1	n= 1117<br>events = 407	1,753 **<br>(1,368 – 2,246)	Reference	1,094<br>(0,809 – 1,479)	1,097<br>(0,813 – 1,480)	1,668 **<br>(1,250 – 2,226)
Model 2	n = 902<br>events = 328	1,811 **<br>(1,356 – 2,418)	Reference	1,232<br>(0,868 – 1,749)	1,238<br>(0,880 – 1,743)	1,760 **<br>(1,256 – 2,466)
Model 3	n = 781<br>events = 289	1,710 *<br>(1,217 – 2,403)	Reference	1,075<br>(0,734 – 1,575)	1,179<br>(0,804 – 1,730)	1,584 *<br>(1,077 – 2,330)
Model 4	n = 780<br>events = 289	1,633 *<br>(1,166 – 2,287)	Reference	1,056<br>(0,720 – 1,547)	1,155<br>(0,788 – 1,693)	1,530 *<br>(1,040 – 2,251)
Model 5	n = 780<br>events = 289	1,676 *<br>(1,186 – 2,368)	Reference	1,061<br>(0,723 – 0,556)	1,169<br>(0,794 – 1,722)	1,552 *<br>(1,048 – 2,299)
Model 6	n= 766<br>events = 282	1,818 *<br>(1,235 – 2,675)	Reference	1,113<br>(0,752 – 1,648)	1,227<br>(0,825 – 1,825)	1,696 *<br>(1,111 – 2,589)
**Cardiovascular death**						
Unadjusted	n = 1110<br>events = 110	1,985 *<br>(1,264 – 3,115)	Reference	0,945<br>(0,530 – 1,685)	1,198<br>(0,694 – 2,068)	1,906 *<br>(1,136 – 3,198)
Model 1	n= 1110<br>events = 110	1,721 *<br>(1,075 – 2,755)	Reference	0,877<br>(0,486 – 1,581)	1,063<br>(0,608 – 1,856)	1,606<br>(0,941 – 2,739)
Model 2	n = 895<br>events = 89	1,357<br>(0,786 – 2,342)	Reference	1,139<br>(0,579 – 2,242)	1,332<br>(0,710 – 2,499)	1,484<br>(0,784 – 2,811)
Model 3	n = 774<br>events = 77	0,958<br>(0,501 – 1,832)	Reference	1,046<br>(0,498 – 2,194)	1,250<br>(0,605 – 2,582)	1,171<br>(0,551 – 2,489)
Model 4	n = 773<br>events = 77	0,927<br>(0,492 – 1,748)	Reference	1,045<br>(0,496 – 2,199)	1,266<br>(0,613 – 2,615)	1,113<br>(0,524 – 2,364)
Model 5	n = 773<br>events = 77	0,955<br>(0,500 – 1,824)	Reference	1,050<br>(0,499 – 2,206)	1,303<br>(0,628 – 2,704)	1,148<br>(0,538 – 2,448)
Model 6	n= 759<br>events = 72	0,709<br>(0,348 – 1,445)	Reference	0,946<br>(0,431 – 2,075)	1,240<br>(0,579 – 2,657)	0,953<br>(0,418 – 2,172)
**MACE**						
Unadjusted	n = 1117<br>events = 428	1,576 **<br>(1,253 – 1,983)	Reference	0,973<br>(0,733 – 1,290)	1,305 *<br>(1,000 – 1,703)	1,477 *<br>(1,129 – 1,933)
Model 1	n= 1117<br>events = 428	1,428 *<br>(1,125 – 1,813)	Reference	0,911<br>(0,683 – 1,213)	1,176<br>(0,895 – 1,544)	1,318<br>(0,999 – 1,738)
Model 2	n = 902<br>events = 344	1,444 *<br>(1,096 – 1,903)	Reference	1,210<br>(0,868 – 1,686)	1,371<br>(0,999 – 1,881)	1,467 *<br>(1,056 – 2,039)
Model 3	n = 781<br>events = 299	1,378 *<br>(1,000 – 1,899)	Reference	1,337<br>(0,926 – 1,931)	1,550 *<br>(1,073 – 2,239)	1,444<br>(0,983 – 2,121)
Model 4	n = 780<br>events = 299	1,342<br>(0,973 – 1,851)	Reference	1,304<br>(0,904 – 1,882)	1,491 *<br>(1,031 – 2,158)	1,372<br>(0,933 – 2,016)
Model 5	n = 780<br>events = 299	1,374<br>(0,989 – 1,909)	Reference	1,311<br>(0,909 – 1,893)	1,514 *<br>(1,043 – 2,198)	1,400<br>(0,947 – 2,070)
Model 6	n= 766<br>events = 290	1,376<br>(0,950 – 1,993)	Reference	1,338<br>(0,915 – 1,957)	1,529 *<br>(1,040 – 2,248)	1,410<br>(0,926 – 2,148)
**Dialysis therapy**						
Unadjusted	n = 1117<br>events = 396	2,390 **<br>(1,876 – 3,044)	Reference	1,310<br>(0,962 – 1,784)	1,920 **<br>(1,434 – 2,570)	2,335 **<br>(1,739 – 3,314)
Model 1	n = 1117<br>events = 396	2,826 **<br>(2,203 – 3,636)	Reference	1,309<br>(0,957 – 1,789)	2,044 **<br>(1,519 – 2,750)	2,693 **<br>(1,990 – 3,644)
Model 2	n = 902<br>events = 325	3,145 **<br>(2,349 – 4,210)	Reference	1,480 *<br>(1,043 – 2,101)	1,948 **<br>(1,388 – 2,734)	3,014 **<br>(2,137 – 4,252)
Model 3	n =781<br>events = 280	1,915 **<br>(1,367 – 2,682)	Reference	1,111<br>(0,756 – 0,1633)	1,207<br>(0,817 – 1,782)	1,730 *<br>(1,165 – 2,569)
Model 4	n = 780<br>events = 280	1,904 **<br>(1,345 – 2,677)	Reference	0,622<br>(0,750 – 1,618)	1,192<br>(0,807 – 1,760)	1,686 *<br>(1,135 – 2,505)

Model 1: Adjusted for age, sex, ethnitcity
Model 2: Additionally adjusted for diabetes mellitus, cardiovascular disease, perioheral vascular disease, chronic heart failure, cerebrovascular disease, myocardial infactation, systolic blood pressure, smoking status and BMI

Model 3: Additionally adjusted for albumin, hemoglobin, potassium, phosphate and presence of ESA oder ACE medication
Model 4: Additionally adjusted for cause of CKD
Model 5: Additionally adjusted for eGFR
Model 6: Additionally adjusted for urea
* p= <0,05 ** p=<0,001
